# The Relationship between Intramuscular Adipose Tissue, Functional Mobility, and Strength in Postmenopausal Women with and without Type 2 Diabetes

**DOI:** 10.1155/2015/872726

**Published:** 2015-01-27

**Authors:** Janet M. Pritchard, Sarah Karampatos, Karen A. Beattie, Lora M. Giangregorio, George Ioannidis, Stephanie A. Atkinson, Lehana Thabane, Hertzel Gerstein, Zubin Punthakee, Jonathan D. Adachi, Alexandra Papaioannou

**Affiliations:** ^1^Geriatric Education and Research in Aging Sciences (GERAS) Centre, St. Peter's Hospital, 88 Maplewood Avenue, Hamilton, ON, Canada L8M 1W9; ^2^Department of Medicine, McMaster University, 1280 Main Street West, Hamilton, ON, Canada L8S 4K1; ^3^Charlton Medical Centre, 25 Charlton Avenue East, Hamilton, ON, Canada L8N 1Y2; ^4^Department of Kinesiology, University of Waterloo, 200 University Avenue West, Waterloo, ON, Canada N2L 3G1; ^5^Department of Pediatrics, McMaster University, 1280 Main Street West, Hamilton, ON, Canada L8S 4K1; ^6^Department of Clinical Epidemiology and Biostatistics, McMaster University, 1280 Main Street West, Hamilton, ON, Canada L8S 4K1

## Abstract

*Objectives*. To determine (1) whether intramuscular adipose tissue (IntraMAT) differs between women with and without type 2 diabetes and (2) the association between IntraMAT and mobility and strength. *Methods*. 59 women ≥ 65 years with and without type 2 diabetes were included. A 1-Tesla MRI was used to acquire images of the leg. Timed-up-and-go (TUG) and grip strength were measured. Regression was used to determine associations between the following: (1) type 2 diabetes and IntraMAT (covariates: age, ethnicity, BMI, waist : hip ratio, and energy expenditure), (2) IntraMAT and TUG (covariates: diabetes, age, BMI, and energy expenditure), and (3) IntraMAT and grip strength (covariates: diabetes, age, height, and lean mass). *Results*. Women with diabetes had more IntraMAT. After adjustment, IntraMAT was similar between groups (diabetes mean [SD] = 13.2 [1.4]%, controls 11.8 [1.3]%, *P* = 0.515). IntraMAT was related to TUG and grip strength, but the relationships became nonsignificant after adjustment for covariates (difference/percent IntraMAT [95% CI]: TUG = 0.041 seconds [−0.079–0.161], *P* = 0.498, grip strength = −0.144 kg [−0.335–0.066], *P* = 0.175). *Conclusions*. IntraMAT alone may not be a clinically important predictor of functional mobility and strength; however, whether losses in functional mobility and strength are promoted by IntraMAT accumulation should be explored.

## 1. Background

Most adults with type 2 diabetes experience accelerated musculoskeletal aging and a higher prevalence of frailty than people without diabetes of a comparable age [1, 2]. This leads to a greater risk of falls and fractures [3], greater healthcare expenditures, and increased morbidity and mortality [4]. Skeletal muscle atrophy and obesity, known as sarcopenic obesity, seem to be important contributing factors but do not entirely explain the variance in functional mobility and strength [5]. Skeletal muscle quality, which is influenced by the infiltration of adipose tissue into skeletal muscle, may also play a role.

Adipose tissue infiltration of the thigh, assessed with the computed tomography- (CT-) derived muscle density measurement [6], is related to lower muscle strength [7] and greater hip fracture risk [8]. However, assessing infiltration of adipose tissue into skeletal muscle with CT is limited. It is an indirect measurement of muscle adiposity and does not differentiate between adipose tissue within the muscle and adipose tissue between the muscle bellies, which may be physiologically different depots of adipose tissue [9]. Compared to CT, studies using magnetic resonance imaging (MRI) are better able to assess adipose tissue within the muscle due to better image resolution and contrast [10]. Various terms have been used to describe these depots of adipose tissue beneath the subcutaneous adipose tissue and fascia of the muscle. For example,* intermuscular adipose tissue *(InterMAT) has been used to describe adipose tissue beneath the fascia and between and within muscle bellies [11–14]. However, others have used the same term to describe adipose tissue beneath the fascia and between the muscle bellies and not within the muscle bellies [15, 16]. Conversely,* intramuscular adipose tissue *(IntraMAT) has been defined by some groups as adipose tissue within the muscle bellies [17–20]. There are limited studies that examine InterMAT and IntraMAT separately using image analysis software, which is important considering that these depots of adipose tissue may have different effects on musculoskeletal health [9, 11].

Functional mobility and muscular strength are important determinants of healthy aging and preserving independence. The time taken to complete a timed-up-and-go (TUG) test is indicative of an older adult's level of function, with higher test scores linked to poor balance, slow gait speed, and difficulty with activities of daily living [21]. Likewise, older adults with poor grip strength are at higher risk of losing independence in activities of daily living [22] and mortality [23, 24]. Both TUG test and grip strength scores have been used to differentiate between frail and nonfrail older adults in the community [25] and are strong independent predictors of physical frailty [26].

The objectives of this study were to (1) determine whether lower leg percent IntraMAT differs between women with and without type 2 diabetes, (2) determine the association between percent IntraMAT and both functional mobility (TUG) and strength (grip strength), and (3) determine whether the relationships are affected by adjusting for type 2 diabetes and other potential covariates.

## 2. Methods

### 2.1. Study Participants

This cross-sectional study was conducted with postmenopausal women of age ≥65 years. Participants were recruited from outpatient diabetes clinics and by community advertisements. Participants with type 2 diabetes had a diagnosis of type 2 diabetes for ≥5 years. This study included participants recruited for the purpose of a study on bone health [27]; therefore exclusion criteria were (1) use of medications in the past 24 months known to affect bone (bisphosphonates, parathyroid hormone, calcitonin, selective estrogen receptor modulator, and hormone therapy); (2) chronic systemic glucocorticoid exposure (≥3 months at a prednisone equivalent dosage ≥ 7.5 mg/day); (3) Paget's disease; (4) hyperparathyroidism or hypoparathyroidism; (5) metastatic cancer in the past 5 years; and (6) severe renal impairment, defined as CrCl < 30 mL/min, which is the National Kidney Foundation cutoff for severe renal impairment or kidney failure [28]. Participants with ferromagnetic implants were excluded from the study. Study approval was granted by the Hamilton Integrated Research Ethics Board.

### 2.2. Magnetic Resonance Imaging (MRI)

**Figure 1:**
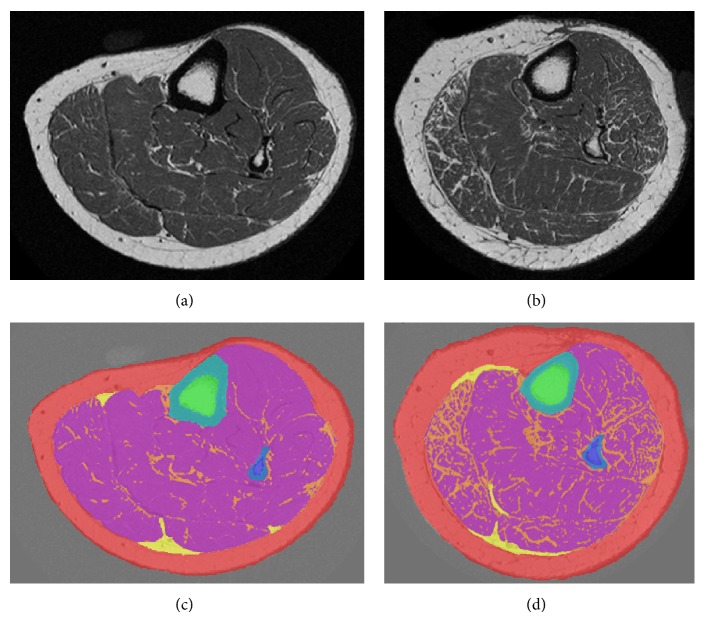
Axial MRI images of the lower leg from a Caucasian postmenopausal woman without type 2 diabetes (age 68 years, percent IntraMAT = 8%) (a) and with type 2 diabetes (age 67 years, percent IntraMAT = 14%) (b). Segmented images of the lower leg from the participant without type 2 diabetes (c) and with type 2 diabetes (d). Subcutaneous adipose tissue (red), muscle (fuchsia), intermuscular adipose tissue (InterMAT) (yellow), intramuscular adipose tissue (IntraMAT) (orange), tibia cortical bone (blue), tibia bone marrow (green), fibula cortical bone (royal blue), and fibula bone marrow (purple).

One researcher imaged each participant's nondominant lower leg with a peripheral 1-Tesla MRI system (OrthOne, GE Healthcare). The standard 66% tibia site (i.e., distance from the distal end of the medial malleolus to the medial knee joint cleft × 0.66) was marked on each participant's leg. Participants inserted their lower leg into a 160 mm diameter transmit/receive coil and padding was applied around the leg to reduce the potential of motion artifact. A 2-dimensional sagittal fast spin echo (FSE) localizer was used to position the participant's leg in the system and ten axial T_1_-weighted images were obtained (Figures 1(a), 1(b)). Scan parameters were as follows: TR = 600 milliseconds (ms), TE = 22.9 ms, gap = 0 mm, flip angle = 90°, bandwidth = 25 kHz, acquisition matrix = 320 × 320, field of view = 16 cm, and scan time = 9:42 minutes.

### 2.3. MRI Analysis

Prior to image analysis, 2 authors (Janet M. Pritchard, Sarah Karampatos) independently reviewed all axial slices for image blurring, and participants with significant motion artifact (graded as 0 for no motion artifact or 1 for motion artifact) were excluded from analysis. Postprocessing of all axial slices was performed using sliceOmatic version 4.3 rev. 7c (Tomovision, Montreal, Canada) (Figures 1(c), 1(d)). One person who was unaware of diabetes status (Sarah Karampatos) completed image analysis on the same workstation. Images were gamma corrected to calibrate the grey level of the images for slice-by-slice segmentation. We segmented the compartments of the lower leg according to the following definitions:

- subcutaneous adipose tissue: any tissue or component outside of the fascia, including vessels and skin;
- total muscle: nonadipose tissue beneath the fascia, excluding bone;
- tibia and fibula bone: cortical bone, excluding marrow;
- intermuscular adipose tissue (InterMAT): adipose tissue beneath the fascia and between the following: (1) soleus and medial head of gastrocnemius, (2) lateral head of the gastrocnemius and medial head of gastrocnemius, and (3) lateral head of gastrocnemius and soleus; if vessels were contained in this compartment, they were included in the segmentation;
- intramuscular adipose tissue (IntraMAT): adipose tissue within the total muscle compartment (belly), including vessels.

The semiautomatic segmentation tools were used to segment the subcutaneous adipose tissue, total muscle, tibia, fibula, and intermuscular adipose tissue. Mathematical morphology was used to compute the watershed of the image and create a water parting mesh of the image. This step distinguishes between tissues with different grey level values, such as muscle and subcutaneous adipose tissue. The images were edited sparingly to refine the segmentation. Region growing was used within the muscle to segment IntraMAT. A threshold was set to exclude pixels belonging to muscle and include pixels belonging to adipose tissue. A slicewise comparison between the original image and segmented image was completed to ensure that the segmentation was sensible. IntraMAT was normalized to the total muscle cross-sectional area (percent IntraMAT = [IntraMAT (mm^2^) ÷ total muscle area (mm^2^)] × 100%). To determine the short-term intrarater reliability of the segmentation protocol, one researcher (Sarah Karampatos) analyzed 21 anonymized randomly selected participants' scans in duplicate [29]. The time between the repeated analyses of a participant's scan was 2 weeks. Previous studies have validated the use of this software for the assessment of adipose tissue within the muscle [30, 31].

### 2.4. Additional Data Collection

Anthropometric, medical history, nutrition, and physical activity data were collected. A whole-body dual X-ray absorptiometry (DXA) (Hologic, Discovery QDR4500A) scan was used to ascertain total body mass, lean mass, and fat mass, from which percent lean and fat mass were derived. Height was measured using a wall-mounted stadiometer and BMI was calculated. Waist and hip circumference were measured to the nearest 0.1 cm. The age-adjusted Charlson Index, a commonly used measure of comorbidity, was calculated for each participant based on the presence of diseases. Weekly energy expenditure in habitual activities was estimated using the modified Paffenbarger Physical Activity Questionnaire, which quantifies kilocalorie (kcal) expenditure based on stair climbing, walking, and participation in activities [32, 33]. Dietary calcium and vitamin D intakes were quantified using a validated food frequency questionnaire [34] and supplemental calcium and vitamin D were captured to calculate total (diet + supplement) intake. Participant's ethnicity was recorded as Caucasian or non-Caucasian.

### 2.5. Functional Mobility and Strength Assessments 

The TUG test was used to assess functional mobility [21]. This objective test has good interrater and intrarater reliability and is correlated with Berg Balance Scale scores, gait speed, and the Barthel Index of activities of daily living (ADLs) [21]. For the TUG test, participants were instructed to stand from a chair, walk 3 meters, turn, walk back to the chair, and sit down [21]. Gait aids were used when needed. To assess muscle strength, an isometric grip strength dynamometer was used (Takei T.T.K 5001 Isometric Grip A Dynamometer, Takei Scientific Instruments). Grip strength is a quick, objective measure of muscular strength and is correlated with quadriceps strength [35]. Grip strength was recorded with the dominant arm to the nearest kilogram for 3 trials, and the average grip strength was calculated for each participant. Arm specific force, a normalized measure of muscle quality, was calculated by taking the ratio of grip strength to arm muscle mass acquired with the DXA whole-body scan [2].

### 2.6. Statistical Analysis

To determine the relative intrarater reliability of the image analysis protocol, type 2,1 intraclass correlation coefficient (ICC) and 95% confidence intervals (CI) were calculated. All data were tested for normality using the Shapiro-Wilk test, and the mean (standard deviation (SD)) or median (interquartile range (25th and 75th percentiles)) was computed. Student's* t*-test was used to determine between-group differences in cross-sectional area of lower leg compartments. To determine whether percent IntraMAT (percent IntraMAT = [IntraMAT (mm^2^) ÷ total muscle area (mm^2^)] × 100%) differed for women with and without type 2 diabetes, unadjusted and adjusted parameter estimates were calculated for percent IntraMAT using an analysis of covariance (ANCOVA) model. Covariates for the adjusted model included age, ethnicity, BMI, waist-to-hip ratio, and energy expenditure in activities, as these variables are related to measures of muscle adiposity [13, 36–38]. Separate linear regression analyses were conducted to determine the relationships between the following: (1) TUG (dependent variable) and IntraMAT (independent variable) and (2) grip strength (dependent variable) and IntraMAT (independent variable). For each dependent variable, three models were produced and included (1) an unadjusted model, (2) a model adjusted for only diagnosis of type 2 diabetes, and (3) a model adjusted for covariates. For the TUG analysis, the covariates included age, diagnosis of type 2 diabetes, BMI, and weekly energy expenditure [39, 40]. For the grip strength analysis, the covariates included age, diagnosis of type 2 diabetes, height, and percent lean mass [2, 41]. The interaction term, IntraMAT^*^diabetes, was removed from the final models once it was shown not to be significant. The coefficient of determination (*R* ^2^) was used to determine the explained variance of the models. Covariates for the ANCOVA and linear regression models were selected on the basis of previous research showing an association with the dependent variable. In addition, all covariates were significantly (*P* < 0.05) related to both the dependent and independent variable in Pearson correlation analyses and had a Pearson correlation coefficient ≥ 0.30 [42]. Linear regression assumptions were checked and met for the models, including (1) linear relationship between dependent and predictor variable, (2) homoscedasticity, (3) normal distribution and independence of errors, and (4) multicollinearity (variance inflation factor ≤ 2). Analyses were performed with SPSS version 20 and an alpha level of ≤0.05 was considered statistically significant.

## 3. Results 

29 participants with type 2 diabetes and 30 participants without type 2 diabetes (controls) completed the study visits. After reviewing the MRI scans for motion, 2 participants' scans were discarded (1 participant with type 2 diabetes, 1 control). Descriptive and anthropometric data are presented in Table 1. Most participants with type 2 diabetes were taking insulin (64% [18/28]) or metformin (39% [11/28]).

### 3.1. Type 2 Diabetes and IntraMAT

**Figure 2:**
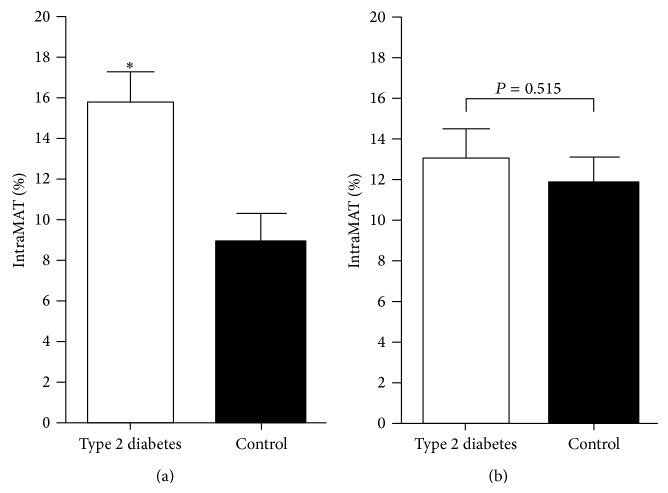
Unadjusted (a) and adjusted (b) comparison of percent IntraMAT in women with and without type 2 diabetes. IntraMAT: intramuscular adipose tissue. ^*^ *P* < 0.05 considered significant. Percent IntraMAT = [IntraMAT (mm^2^) ÷ total muscle area (mm^2^)] × 100%. Covariates: age, ethnicity, BMI, waist : hip ratio, and weekly energy expenditure.

Unadjusted analyses revealed that the absolute amount of IntraMAT was greater in women with type 2 diabetes compared to controls, and there was no between-group difference in the amounts of InterMAT, subcutaneous adipose tissue, total muscle, or bone (Table 2). Unadjusted percent IntraMAT was greater in women with type 2 diabetes (mean [SD], 15.8 [1.5]%) compared to controls (8.9 [1.4]%, *P* = 0.002) (Figure 2(a)); however, after adjustment for age, ethnicity, BMI, waist : hip ratio, and energy expenditure, the between-group difference was reduced (13.2 [1.4]% versus 11.8 [1.3]%, *P* = 0.515) (Figure 2(b)).

### 3.2. Relationship between IntraMAT and Functional Mobility and Strength

Results of the linear regression models are presented in Table 3. There was a significant relationship between IntraMAT and TUG, where IntraMAT alone explained 18.9% of the variance in TUG result (Table 3, *P* = 0.001). Independent of type 2 diabetes, IntraMAT was still related to TUG (*P* = 0.019). After adjusting for type 2 diabetes, age, BMI, and energy expenditure, there was no statistically significant independent relationship between IntraMAT and TUG (*P* = 0.378). The model including IntraMAT, type 2 diabetes, age, BMI, and estimated energy expenditure explained 50.5% of the variance in TUG result (*P* < 0.001). There was no interaction between IntraMAT and diabetes (*P* = 0.754).

There was an inverse relationship between IntraMAT and grip strength, where IntraMAT alone explained 11.2% of the variance in grip strength (Table 3, *P* = 0.012). After adjusting for diagnosis of type 2 diabetes, the relationship between IntraMAT and grip strength remained significant (*P* = 0.049). Adjustment for type 2 diabetes, age, height, and percent lean mass revealed no statistically independent relationship between IntraMAT and grip strength (*P* = 0.120). There was no interaction between IntraMAT and diabetes (*P* = 0.993). The model including IntraMAT, type 2 diabetes, age, height, and percent lean mass explained 28.8% of the variance in grip strength (*P* < 0.001).

### 3.3. Intrarater Reliability

The intrarater reliability for the image analysis protocol was acceptable for segmentation of each compartment. The ICC estimates were as follows: IntraMAT ICC = 0.999 (95% CI, 0.997–0.999, *P* < 0.001), InterMAT ICC = 0.981 (95% CI, 0.953–0.992, *P* < 0.001), subcutaneous adipose tissue ICC = 0.999 (95% CI, 0.997–1.00, *P* < 0.001), total muscle ICC = 0.994 (95% CI, 0.986–0.998, *P* < 0.001), tibia ICC = 0.994 (95% CI, 0.986–0.998, *P* < 0.001), and fibula ICC = 0.972 (95% CI, 0.932–0.989, *P* < 0.001).

## 4. Discussion

This study demonstrated that women with type 2 diabetes have more IntraMAT in the lower leg compared to women without type 2 diabetes, according to the unadjusted analysis. However, adjustment for potential covariates attenuated this difference, suggesting that there may be other factors, such as greater age, ethnicity, BMI, waist : hip ratio, and energy expenditure, which contributed to the difference in IntraMAT between groups. Regarding functional mobility and strength, unadjusted analyses showed that, for each 5% increase in IntraMAT, there was a 1-second increase in TUG result and approximately 1 kg decrease in grip strength. However, following adjustment for potential covariates, a 5% increase in IntraMAT only translated to a 0.20-second increase in TUG result and a 0.70 kg decrease in grip strength, and these associations were not statistically significant. This study also revealed that when considering IntraMAT in disease states, such as type 2 diabetes, covariates should also be considered and that IntraMAT alone is not strongly associated with functional mobility and strength.

This study sought to determine whether percent IntraMAT in the lower leg was different among women with and without type 2 diabetes, as IntraMAT may be one of the mediating factors responsible for the accelerated musculoskeletal aging observed in people with type 2 diabetes [1]. In agreement with other studies [2, 43, 44], we found that our cohort of women with type 2 diabetes had greater TUG test scores indicating poorer functional mobility, lower grip strength, and lower specific force, an indirect measure of muscle quality. Various mechanisms for poor functional mobility and strength in adults with type 2 diabetes have been proposed and include reduced muscle fibre size and number [45] and reduced *α*-motor neuron innervation [46]; however we hypothesized that IntraMAT might also be a likely mediator. On the microscopic level, intramyocellular lipid inhibits skeletal muscle oxidative phosphorylation ability [36, 47], and inflammatory cytokines secreted by adipose tissue in the skeletal muscle microenvironment [48] may lead to proteolysis and muscle catabolism [49]. Previous studies investigating muscle adiposity in adults with type 2 diabetes have used CT to indirectly assess muscle adiposity [37] or did not separate the depots of adipose tissue within the muscle from adipose tissue between the muscle bellies [12, 15, 17]. One study did elegantly separate the adipose tissue within the muscle bellies and adipose tissue between muscle bellies using MRI and also investigated diabetes-related differences in these compartments [17]. However, the authors reported no difference in the absolute amount of adipose tissue within muscle bellies in women with type 2 diabetes compared to controls [17], whereas we did find that women with type 2 diabetes had more IntraMAT in the lower leg. The discrepancy in results may be due to the fact that the mean age and BMI of participants with type 2 diabetes were lower compared to our study cohort, which may have blunted the difference in IntraMAT between groups, as IntraMAT is related to age and BMI [36]. Given that our image segmentation technique differentiates between the adipose tissue within the muscle bellies (IntraMAT) and that between the muscle bellies (InterMAT), two depots of adipose tissue which may be physiologically distinct, our findings may be used as rationale for further noninvasive studies on whether exercise interventions modify IntraMAT in adults with type 2 diabetes and whether modification results in improved metabolic control of diabetes, functional mobility, and strength. It may be important to consider other variables, such as age, ethnicity, energy expenditure in activities, and anthropometrics, as these variables blunted the association between IntraMAT and type 2 diabetes. However, it should be noted that lower energy expenditure, obesity, and abdominal adiposity are common characteristics of adults with type 2 diabetes.

Similar to other studies using MRI, our unadjusted analysis revealed that IntraMAT is related to functional mobility and strength [14, 18]. After adjusting for diabetes, the relationships were weakened but remained significant suggesting that this relationship is not unique to older adults with type 2 diabetes and rather may be a characteristic of aging. This may be explained in part by the idea that IntraMAT may diminish the activation of skeletal muscle, thus worsening functional mobility and strength [19]. Our study makes an important contribution to the literature because, unlike previous studies that have examined the relationships between muscle adiposity, function, and strength [14, 18], our analyses included potential covariates. In the study by Tuttle and colleagues, 78% of study participants had type 2 diabetes and 69% of the participants with type 2 diabetes had peripheral neuropathy [14], findings that were unaccounted for in the analysis making it difficult to conclude whether the reported relationships between muscle adiposity, function, and strength were driven by diabetes or neuropathy. In addition, the image segmentation techniques that were employed in both studies did not clearly distinguish between adipose tissue within muscle bellies and that between muscle bellies. Our study suggests that if IntraMAT is used as an outcome in future studies, investigators should consider possible confounding factors such as age, BMI, and energy expenditure in activities when assessing TUG as the dependent variable and age, height, and percent lean mass when assessing grip strength as the dependent variable. While IntraMAT alone was not strongly associated with functional mobility and strength in this study, larger studies should investigate whether assessing IntraMAT in conjunction with measures of sarcopenia, such as appendicular lean mass, would help clinicians discriminate those who are at risk of declines in functional mobility and muscular strength and may benefit from pharmacologic or lifestyle intervention. Promising results have been published suggesting that aerobic and eccentric resistance training may reduce the amount of IntraMAT and increase lean mass in the thigh and improve functional outcomes in older adults [50].

There are study limitations to acknowledge. First, participants in this study were recruited for the purposes of a study on skeletal health, and participants with diabetes were recruited from an outpatient clinic. Therefore, exclusion criteria were specific to that study and selection bias may have occurred, limiting the generalizability of our findings. Second, the study was powered to examine trabecular bone hole size in women with and without type 2 diabetes [27]. Future adequately powered studies should be conducted to confirm our results. Third, although the Paffenbarger Physical Activity Questionnaire has been validated [33, 51], we recognize that participants tend to overestimate physical activity levels by questionnaire, and assessment by accelerometer is superior. Fourth, peripheral neuropathy was not assessed for this study, and previous studies have suggested that adults with type 2 diabetes and peripheral neuropathy have more IntraMAT compared to controls [15]. Fifth, while others have validated the use of sliceOmatic software for the quantification of muscle adiposity, we have not yet validated our own method against a gold standard [30, 31]. Finally, due to the cross-sectional nature of our study, we were not able to determine whether IntraMAT causes poor functional mobility or strength. A longitudinal analysis of IntraMAT, functional mobility, and strength changes may help discern the directionality of the relationships.

## 5. Conclusion

In conclusion, our study makes a unique contribution to research examining IntraMAT, diabetes status, functional mobility, and strength because we employed an image segmentation methodology that differentiated between adipose tissue within the muscle bellies (IntraMAT) and that between the muscle bellies (InterMAT) and accounted for potential confounders in multiple linear regression analyses. While the unadjusted analysis demonstrated that women with type 2 diabetes have more IntraMAT within the muscle bellies, after considering other factors that could influence IntraMAT, women with type 2 diabetes did not have more IntraMAT compared to women without diabetes. In addition, IntraMAT alone is not associated with functional mobility and strength but in combination with other covariates may serve as a noninvasive biomarker of physical frailty in older adults.

## Figures and Tables

**Table 1:** Characteristics of study participants.

	Type 2 diabetes *n* = 28	Control *n* = 29	*P* value
Age, years	70.0 (68.0–74.0)	70.0 (68.0–73.5)	0.885
Years since diabetes diagnosis, years	17 (11)	—	—
Caucasian, *n* (%)	23 (82.1)	29 (100.0)	0.023^*^
Uses walking aid, *n* (%)	6 (21.4)	2 (6.9)	0.115
BMI, kg/m^2^	34.3 (7.5)	28.0 (5.6)	<0.001^*^
Waist : hip ratio	0.89 (0.07)	0.83 (0.06)	0.005^*^
Percent body fat, %	39.3 (36.5–45.0)	38.5 (34.3–41.7)	0.693
Percent lean mass, %	57.9 (53.1–61.2)	60.8 (55.8–63.6)	0.550
Energy expenditure^a^, kcal/week	1003 (302–2120)	1597 (1129–3865)	0.684
Age-adjusted Charlson Index	4.4 (1.6)	0.4 (1.3)	<0.001^*^
Number of years since menopause	20 (20–25)	20 (19–26)	0.857
Number of prescribed medications	6 (3)	2 (2)	<0.001^*^
Total calcium intake, mg/day	1594 (709)	2048 (589)	<0.001^*^
Total vitamin D intake, IU/day	794 (631)	1204 (938)	0.062
Current smoker, *n* (%)	2 (7.1)	0	0.237
Hip or knee osteoarthritis, *n* (%)	15 (53.6)	5 (17.2)	0.006^*^
TUG, seconds	11.8 (6.2–17.4)	9.0 (6.0–12.0)	<0.001^*^
Grip strength, kg	18.8 (4.9)	21.5 (6.3)	0.059
Specific force, kg_force_/kg_arm mass_	7.5 (2.7)	10.1 (3.4)	0.002^*^

Data are mean (SD) or median (25th–75th percentiles) unless indicated otherwise.

^*^ *P* < 0.05 considered significant.

BMI: body mass index; TUG: timed-up-and-go.

^a^Energy expenditure based on habitual stair climbing, walking, and participation in recreational activities.

**Table 2:** Unadjusted comparison of cross-sectional area of lower leg compartments in women with and without type 2 diabetes.

	Type 2 diabetes *n* = 28	Control *n* = 29	*P* value
IntraMAT, mm^2^	608.2 (463.5–1131.7)	393.4 (282.4–506.1)	0.012^*^
InterMAT, mm^2^	96.8 (66.9–175.8)	80.6 (55.4–148.9)	0.146
Subcutaneous adipose tissue, mm^2^	3724.9 (1587.3)	3538.6 (1156.1)	0.614
Total muscle, mm^2^	5120.5 (845.2)	5072.3 (780.8)	0.824
Tibia, mm^2^	302.5 (52.0)	289.6 (37.7)	0.285
Fibula, mm^2^	64.9 (14.7)	60.1 (13.1)	0.202

Data are mean (SD) or median (interquartile range). ^*^ *P* < 0.05 considered significant.

IntraMAT: intramuscular adipose tissue; InterMAT: intermuscular adipose tissue.

**Table 3:** Unadjusted and adjusted relationships among IntraMAT, functional mobility, and strength.

	Unadjusted			Adjusted for diabetes status			Adjusted for other covariates		
	Incremental difference per percent IntraMAT (95% CI)	*P* value	*R* ^2^	Incremental difference per percent IntraMAT (95% CI)	*P* value	*R* ^2^	Incremental difference per percent IntraMAT (95% CI)	*P* value	*R* ^2^
TUG (sec)	0.188 (0.082 to 0.295)^*^	**<0.001**	0.189^*^	0.137 (0.023 to 0.250)^*^	**0.019**	0.219^*^	0.048 (−0.061 to 0.158)^a^	0.378	0.505^*^
Grip strength (kg)	−0.225 (−0.400 to −0.051)^*^	**0.012**	0.112^*^	−0.193 (−0.385 to −0.001)^*^	**0.049**	0.123^*^	−0.145 (−0.328 to 0.039)^b^	0.120	0.288^*^

^*^ *P* < 0.05 considered significant.

IntraMAT: intramuscular adipose tissue.

^a^Adjusted model including the following covariates: type 2 diabetes, age, BMI, and energy expenditure.

^b^Adjusted model including the following covariates: type 2 diabetes, age, height, and percent lean mass.
